# Phylogenetic Analysis of Porcine Epidemic Diarrhea Virus (PEDV) during 2020–2022 and Isolation of a Variant Recombinant PEDV Strain

**DOI:** 10.3390/ijms252010878

**Published:** 2024-10-10

**Authors:** Qianling Peng, Ping Fu, Yutong Zhou, Yifei Lang, Shan Zhao, Yiping Wen, Yiping Wang, Rui Wu, Qin Zhao, Senyan Du, Sanjie Cao, Xiaobo Huang, Qigui Yan

**Affiliations:** 1 Swine Disease Research Center, College of Veterinary Medicine, Sichuan Agricultural University, Chengdu 611130, China; 2 Key Laboratory of Animal Disease and Human Health of Sichuan Province, Sichuan Agricultural University, Chengdu 611130, China

**Keywords:** PEDV, phylogeny, virus isolation, variant, recombination

## Abstract

Porcine epidemic diarrhea (PED) is an acute, highly contagious, and infectious disease caused by porcine epidemic diarrhea virus (PEDV). PEDV can affect pigs of all ages, with 50~100% mortality in neonatal piglets and substantial economic losses in the swine industry. In the present study, 347 fecal and intestinal samples were collected from seven regions in China during 2020–2022. A comprehensive molecular investigation of the spike (S) gene of PEDV strains was carried out, which included phylogenetic analysis of the obtained PEDV sequences. Epidemiological surveillance data indicate that the GIIc subgroup strains are widely distributed among pigs. A PEDV strain was successfully isolated from positive small intestine samples and identified through RT-PCR detection using specific N gene primers of PEDV, indirect immunofluorescence assay (IFA), TEM analysis, genome sequencing, and full-length S gene analysis, named PEDV/SC/2022. RDP and SimPlot analysis showed that the isolate originated from the recombination of PEDV/AH2012 and PEDV/AJ1102. In conclusion, our findings contribute to the current understanding of PEDV epidemiology and provide valuable information for the control of PED outbreaks in China.

## 1. Introduction

Porcine epidemic diarrhea virus (PEDV) was first detected in the intestinal contents of pigs in Belgium in 1978 [1], and subsequently spread to Japan, China, South Korea, and other neighboring Asian countries [2]. PEDV belongs to the *Alphacoronavirus* genus in the subfamily Coronavirinae of the family *Coronaviridae* [3,4], together with transmissible gastroenteritis virus (TGEV) [5], human coronavirus-229E (HCoV-229E) [6], and canine coronavirus (CCoV) [7]. PEDV is an enveloped, single-stranded, positive-sense RNA virus with a genome of approximately 28 kb in length. The genome of PEDV is divided into three open reading frames (ORFs): replicase ORF1a and ORF1b, translated to nonstructural proteins; ORFs encoding structural proteins; and ORFs encoding accessory proteins [8]. PEDV comprises four structural proteins, namely spike (S), nucleocapsid (N), membrane (M), and envelop (E) proteins, in addition to sixteen nonstructural proteins (nsp1–16) and an accessory protein, ORF3 [9].

This acute, highly contagious, and infectious disease, named PED, can be caused by PEDV and is characterized by watery diarrhea and vomiting in piglets, followed by rapid dehydration and death. PED can result in high morbidity and mortality in neonatal piglets, as well as serious economic losses to the swine industry. In China, PEDV was first isolated in 1984, which led to sporadic circulation in the swine population, without large-scale outbreaks [10,11,12]. In 2010, highly virulent PEDV strains emerged in China, causing severe PEDV outbreaks that subsequently spread across the country [10,13,14,15].

PEDV can be classified into two genotypes (GI and GII), based on the complete genomes, and can be further subdivided into the GIa, GIb, GIIa, and GIIb subgroups [16]. The GIa subgroups include classic strains, whereas the strains of the GIb subgroups were the first identified in China [14]. Compared to Chinese GIb strains, strains from the United States have insertions and deletions in the S gene, also known as S-Indel strains [17]. The GII genotype includes variant strains that have emerged since 2011 [18]. While the GIIa subgroup strains originated in the United States, the GIIb subgroups strains and some GIIa subgroups strains originated in Asia [12,19].

In recent years, many studies have shed light on the molecular epidemiology and evolutionary dynamics of PEDV, revealing the high prevalence of GII subgroups in China [18,20,21,22,23,24,25]. To further understand its genetic evolution and diversity, a detailed prevalence investigation of PEDV was conducted from phylogenetic perspectives. Clinical samples were selected from regions of Sichuan Province. This study illustrates the genetic diversity of PEDV and isolates a recombinant PEDV strain. We elucidate its molecular characteristics and phylogenetic information. The results obtained can contribute to paving the way for predicting the prevalence and prevention of PEDV by analyzing the characteristics of novel strains.

## 2. Results

### 2.1. Prevalence of PEDV in Clinical Samples from Diarrheal Pigs

A total of 347 porcine diarrheal samples (including feces and intestine tissues) were collected from seven regions in China during 2020–2022 for PEDV detection. The overall positive rate was 46.39% (Table 1).

**Figure 1:**
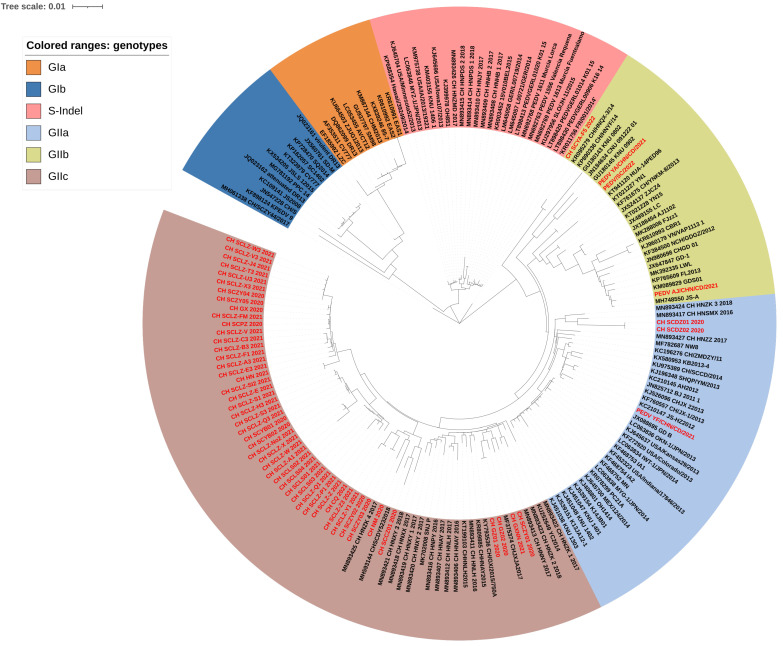
ML trees of the PEDV S gene. Scale bar: 0.01 (model: GTR + G + I). The sequences collected in this study were marker in red.

**Figure 2:**
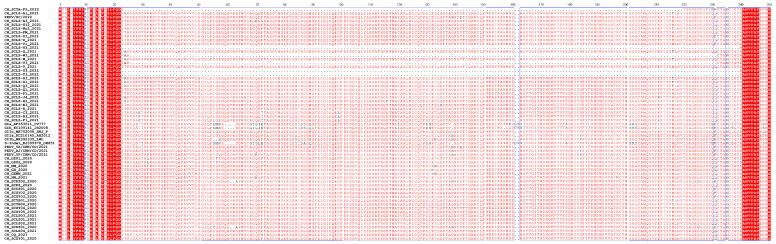
Amino acid sequence alignment of the PEDV S gene (aa 1–250). The first row represents the site of the amino acid. The black letters represents the name of the sequences. The conserved amino acid sites are marked with the red background and blue boxes.

From the 199 positive samples, 55 full-length S genes were obtained and then subjected to further phylogenetic analysis alongside 119 reference strains. The ML tree of PEDV illustrated that these sequences could be classified into six groups: GIa, GIb, S-Indel, GIIa, GIIb, and GIIc subgroups, consistent with previous reports [19,24]. Specifically, the ML tree (Figure 1) revealed that 3 strains were clustered in the GIIa subgroup, 4 strains in the GIIb subgroup, and 48 strains in the GIIc subgroup. The results indicate that the GIIc strains are widely distributed in China. In order to investigate the changed essential residues in various PEDV subgroups, amino acid sequence alignments of the amino acid nucleotides were carried out (Figure 2).

### 2.2. Virus Isolation and Identification

**Figure 3:**
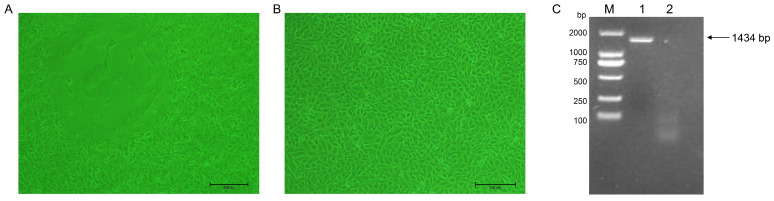
The results of PEDV-infected Vero cells. (**A**) PEDV-infected Vero cells (100×). (**B**) Normal Vero cells (100×). (**C**) Amplification results of RT-PCR. M: DNA Marker 2000. 1: PEDV N gene. 2: Negative control.

PEDV-positive samples were processed and used for virus isolation on Vero cells as previously described. An evident typical CPE caused by PEDV was observed after seven blind passages. In comparison to the uninfected Vero cells, the CPE that appeared in the PEDV-infected Vero cells was characterized by cell fusion, contraction, and syncytial formation (Figure 3A,B). The successful isolation of PEDV was confirmed by the RT-PCR results, identifying the strain as PEDV/SC/2022 (Figure 3C).

**Figure 4:**
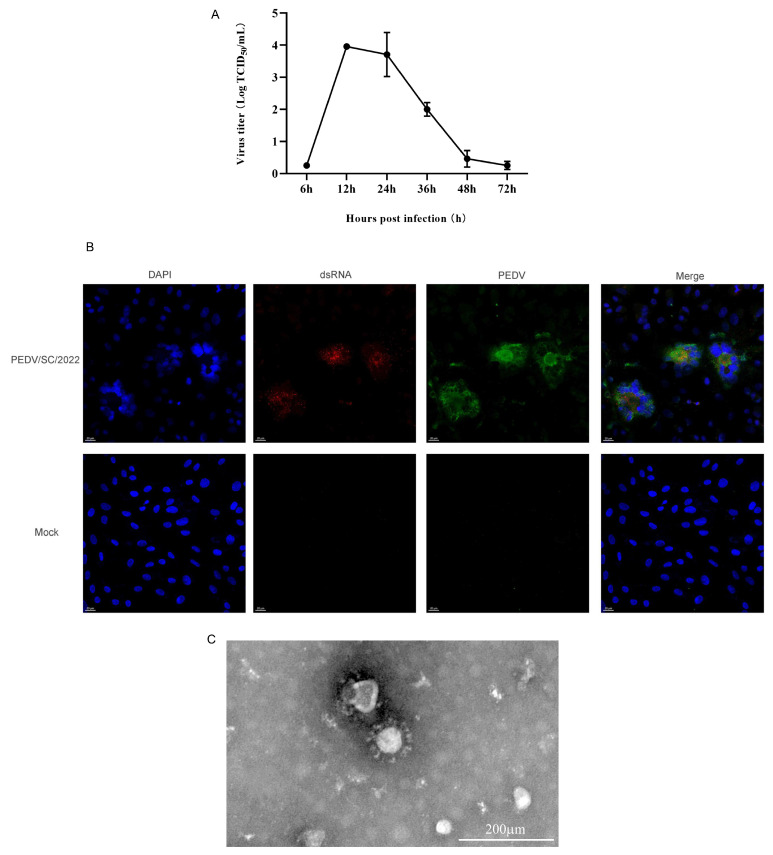
PEDV isolate replicated in Vero cells. (**A**) Growth curve of PEDV isolate at in Vero. Data are presented as mean ± SD of triplicates. (**B**) Detection of PEDV infection in Vero cells by IFA. Cells were immunostained for PEDV (green) and dsRNA (red). Nuclei were stained with DAPI (blue). (**C**) Electron micrograph of purified isolate negatively stained with 2% phosphotungstic acid (×50 K).

The viral titer of PEDV/SC/2022 was determined at different timepoints, reaching a peak of 10^4^ TCID_50_/mL at 12 hpi (Figure 4A). The IFA results demonstrate that PEDV/SC/2022 specifically bound to the PEDV-positive serum, while no specific fluorescence was observed in the non-inoculated cells (Figure 4B). The specific byproduct of viral replication, double-strand RNA (dsRNA), was also observed (Figure 4B). In the TEM analysis of the negative stained samples, the viral particles appeared to be spherical, with an average diameter of around 100 nm, exhibiting spikes on the membrane and resembling a crown-like structure typical of coronavirus (Figure 4C).

### 2.3. Phylogenetic Analysis and Alignment of the Genome and S Gene of PEDV/SC/2022

**Figure 5:**
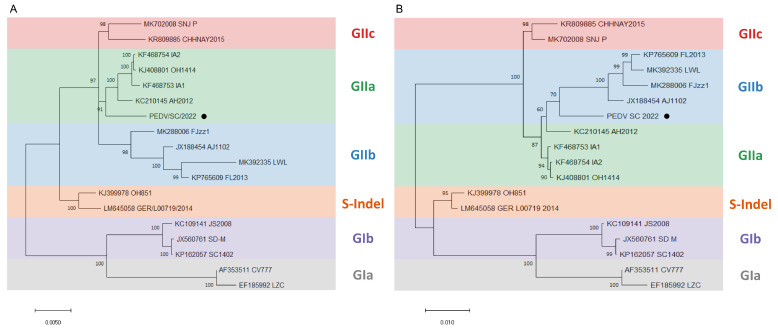
ML trees based on the nucleotide sequences of the whole genome (**A**) and full-length S gene (**B**) of the PEDV isolate in this study, and other representative strains. The black circle represents the isolate in this study.

To perform the phylogenetic analysis, an ML tree was constructed based on the genome nucleotide sequences of PEDV/SC/2022 and 17 classic PEDV strains from GenBank. The information of the 17 classic PEDV strains is listed in Table 2. The ML tree of the genome revealed that PEDV/SC/2022 belongs to the GIIa subgroup and shows a close relationship with PEDV/AH2012 (Figure 5A). However, according to the ML tree of the S gene, PEDV/SC/2022 clustered with the GIIb subgroup and was closely related to PEDV/AJ1102 (Figure 5B).

**Figure 6:**
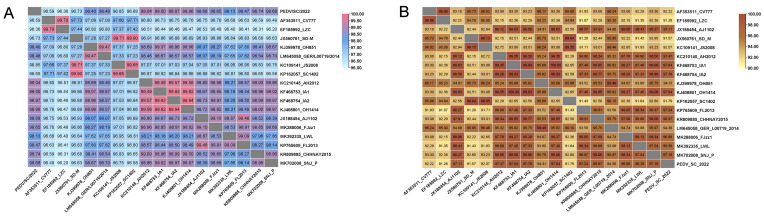
Nucleotide sequence homology analysis of the whole genome (**A**) and full-length S gene (**B**) of the PEDV isolate in this study, and other representative strains.

The nucleotide homology between PEDV/SC/2022 and the 17 representative strains ranged from 96.36% to 99.04%, with the highest similarity with PEDV/AH2012 (Figure 6A). The amino acid homology ranged from 91.57% to 97.84%, showing high similarity to the GIIa strains (AH2012:97.47%, IA1: 97.84%, IA2: 97.76%, and OH1414: 97.84%) and the GIIb strains (AJ1102: 97.11%) (Figure 6B).

### 2.4. Recombination within the PEDV/SC/2022 ORF1b Gene

**Figure 7:**
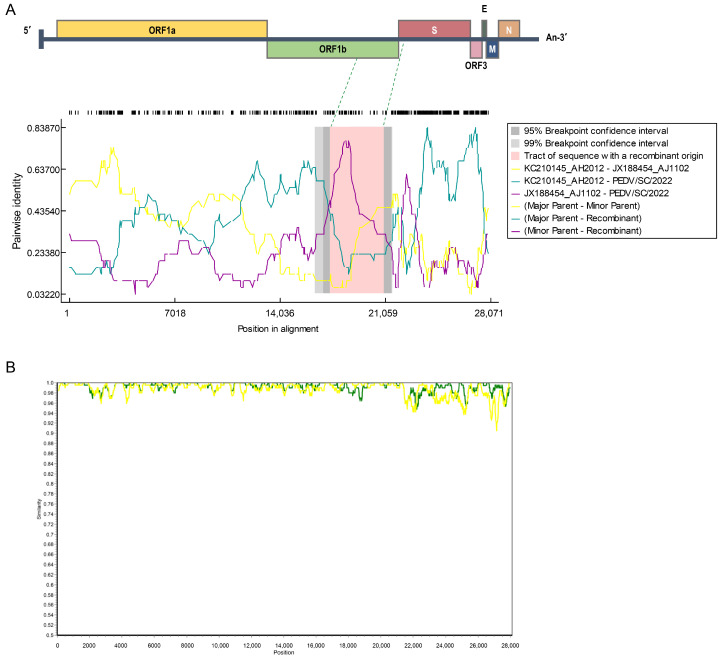
Recombination analysis of PEDV strains. The results of RDP4 are supported by ≥6 programs (**A**). The Y-axis shows the pairwise identity, and the X-axis indicates the positions in alignment. The dotted green line in (**A**) indicates the regions where recombination events may occur. The breakpoint was identified with SimPlot (**B**). The green and yellow lines in (**B**) represents KC21014_AH2012 and JX188454_AJ1102, respectively.

To further determine the associations among the PEDV/SC/2022, AH2012, and AJ1102 strains, recombination analysis was performed using RDP4. As shown in Figure 7A, a recombination event was detected, with the major parent strain identified as AH2012 and the minor parent strain as AJ1102. The potential recombinant breakpoints were identified (nt 17,414–20,966), situated at the ORF1b and S region. These results are supported by the SimPlot analysis results, as shown in Figure 7B, suggesting that PEDV/SC/2022 might have evolved from a natural recombination between the classical GIIa and GIIb strains.

## 3. Discussion

At present, PEDV has emerged as a significant and highly contagious pathogen in the swine industry. In 2010, DR13-like PEDV strains have extensively spread throughout China and have become the major pathogen of swine viral diarrhea disease [14]. The vaccine developed from the classic strain CV777 has been proven ineffective in protecting pigs in China [26]. In this study, 347 samples from seven regions (Sichuan, Guizhou, Chongqing, Inner Mongolia, Henan, Jiangsu, and Guangxi) were collected during 2020–2022. The samples were detected via RT-PCR detection using specific N gene primers of PEDV; the PEDV-positive rate is presented in Table 1. The infection rate of PEDV was found to be similar to that in previous reports [21,23,27,28,29,30], indicating common occurrences of PEDV infections in China’s swine industry.

To further study the evolution features of PEDV, PCR amplicons of the S genes of 55 samples were analyzed with 119 reference strains. Out of the 55 PEDV strains analyzed in this study, only 3 genotypes were identified as PEDV GIIa, GIIb, and GIIc. The prevalent rates of each were 5.45% (3/55), 7.27% (4/55), and 87.28% (48/55), respectively. Among the 55 strains collected in this study, 48 were from Sichuan Province, with 35 sequences clustering with GIIc, with 4 with GIIb, and 3 with GIIa. These results align with the prevalence of PEDV in Sichuan Province from 2014 to 2018 [31], indicating that PEDV-GIIc is the predominant strain in Sichuan Province, China. While our findings provide insight into the prevalence in Sichuan Province, the findings cannot accurately depict the overall prevalence of PEDV in Guangxi, Henan, Inner Mongolia, Chongqing, and Guizhou Provinces due to the limited sample size. The sequences of PEDV obtained in the study are primarily clustered with those from Henan Province. It is estimated that this may be attributed to live swine trade within China. A prior retrospective study suggested that Henan and Guangdong Provinces are primary hubs for PEDV spread [32]. In the past few years, several reports have investigated the prevalence rates of PEDV in Henan Province, with positive rates above 50% [33,34,35]. Geographically, Henan Province maintains connections with most other provinces. At the same time, Henan Province is the largest province in China for live swine trade, leading in the transportation of pigs. The movement of live animals with trade networks, contaminated fomites, and human activities represent potential pathways for virus transmission across diverse farms, regions, and even countries. Therefore, the transportation of live swine from Henan to Sichuan Province could be the main factor leading to the clustering of collected strains with those obtained from Henan Province.

As the primary structural protein of PEDV, amino acid sequences of the spike protein obtained in this study were compared to several classic reference strains. Six sequences exhibited continuous deletion in the N-terminal domain (NTD), which were CH_SCLZ-T3_2021 (deletion at aa 23–239), CH_SCLZ-U3_2021 (deletion at aa 23–239), CH_SCLZ-Z3_2021 (deletion at aa 23–239), CH_SCLZ-V3_2021 (deletion at aa 29–234), CH_SCLZ-W3_2021 (deletion at aa 29–234), and CH_SCYA_F5_2022 (deletion at aa 25–220). The main functions of the S protein are cell receptor binding, membrane fusion mediating, and inducing neutralizing antibody production [36,37]. Therefore, it is hypothesized that these deletions may alter the tropism, pathogenicity, and antigenicity of this coronavirus.

Previous reports have shown that porcine respiratory coronavirus (PRCV), the deficient mutant of TGEV, results from the deletion of aa 21–244 in the NTD of TGEV, which leads to alterations in the virus tropism and pathogenicity, resulting in its mutation into PRCV [38]. The S protein of the MF3809/2008/South Korea strain was continuously deficient in aa 713–916, which is located in the CTD of S1 and the NTD of S2, resulting in the destruction of four glycosylation sites and two neutralization epitopes [39]. This could lead to alternations in the antigenicity and immunogenicity of the MF3809/2008/South Korea strain. Also, deletions in the spike protein can alter the virulence of PEDV strains. A non-lethal PEDV strain, Tottori2, was identified by Masuda T et al. in 2014, who demonstrated that the deletions in the S gene (582 nt) could affect its virulence [40]. The PEDV strain PC21A, which is the culture-adapted PC177 (TC-PC177), contains a 197-aa deletion in the NTD of the S gene, also known as S1 NTD-del PEDV. Hou found that TC-PC177 only caused moderate diarrhea and no mortality in neonatal pigs [41]. However, it was found by Su et al. that the S1 NTD-del PEDV might facilitate effects on the infection of S-intact PEDV during co-infection [42]. Accordingly, epidemiological investigation, phylogenetic analysis and molecular characterization of PEDV-prevalent strains are crucial for the prevention and control of it, considering the diversity of PEDV S genes and the complexity of the current PEDV epidemic situation.

To better control the ongoing PED epidemic in China, there is a need for the isolation and identification of newly variant PEDV strains. Vero cells, commonly used to isolate PEDV since it was first discovered, have been subject to various methods to isolate the virus. The addition of exogenous trypsin is essential for the isolation of wild-type PEDV strains, as the S protein must be cleaved by exogenous trypsin into two subunits, S1 and S2, promoting receptor recognition and membrane fusion between the virus and cells. The presence of trypsin can promote the release of viruses from the cell surface, enhance PEDV infection, and facilitate the efficient spread of PEDV to neighboring cells [43]. In 1988, Hofmann’s study demonstrated for the first time that PEDV can adapt in Vero cell cultures by adding trypsin to the medium [44].

In this study, an appropriate amount of trypsin was added to the medium during the virus isolation process to increase the success rate. After several blind passages, CPE such as cell fusion and syncytia formation were observed after passage 7 in the Vero cells. By RT-PCR amplification, IFA, TEM analysis, infectious virus titer determination, and growth kinetics determination, it was demonstrated that the isolate (PEDV/SC/2022) was phenotypically stable. To characterize PEDV/SC/2022, the whole genome and spike gene were successfully sequenced. PEDV/SC/2022 belonged to the GIIa subgroup according to the ML tree based on the whole genome, closely related to AH2012. However, when PEDV/SC/2022 was analyzed based on the S gene, it was closely related to the GIIb strain, AJ1102. Subsequently, the results of RDP4 revealed that PEDV/SC/2022 may be a recombinant strain derived from AH2012 and AJ1102. PEDV/AH2012 was initially isolated in Anhui Province, China [44], whereas PEDV/AJ1102 was isolated in Hubei Province, China [45]. Furthermore, PEDV/AJ1102 has already been used in commercial vaccines. PEDV/SC/2022, a recombinant strain isolated in Sichuan Province, was derived from two strains from different provinces. A previous phylogeographic study indicated that Hubei Province may have caused new introductions to nearby provinces, mostly in the west of China, such as Anhui Province [32]. We consider that the mixed infection or re-infections of vaccinated pigs may result in the emergence of new strains. SimPlot further identified that the breakpoint is located in the ORF1b region (17,414–20,966 nt). ORF1b encoded nonstructural proteins 12–16, involved in virus replication and genome packaging [45]. Recombination in this region may be decisive for the viral viability and transmissibility. The emergence of PEDV/SC/2022 suggests that the use of the commercial PEDV vaccine not only fails to provide complete and effective protection against prevalent variant strains, but also might act as an evolutionary catalyst, leading to the emergence of highly virulent strains.

## 4. Materials and Methods

### 4.1. Cells, Antibodies, and Clinical Samples

Vero cells, preserved by the College of Veterinary Medicine, Sichuan Agricultural University, were cultured in Dulbecco’s modified Eagle medium (DMEM; Gibco, Grand Island, NY, USA) supplemented with 10% (*w*/*v*) fetal bovine serum (FBS) (ExCell Bio, Suzhou, China).

The antibodies used in this study included fluorescein isothiocyanate (FITC)-conjugated goat anti-pig immunoglobulin G (IgG) and cyanine 3 (Cy3)-conjugated goat anti-mouse IgG, used in an immunofluorescence assay (IFA) purchased from Abcam (Cambridge, UK). Mouse anti-double stranded RNA was purchased from SCICONS (Szirák, Hungary).

From 2020 to 2022, a total of 347 samples were collected from pigs with diarrhea in seven regions (Sichuan, Chongqing, Guizhou, Inner Mongolia, Henan, Jiangsu, and Guangxi) in China, comprising 200 small intestine tissue samples and 147 fecal samples. All samples were stored at −80 °C prior to utilization. 

### 4.2. RNA Extraction and Detection of PEDV

Prior to RNA isolation, the tissue samples were homogenized in sterile phosphate buffer saline (PBS) and then centrifuged at 185× *g* for 5 min after undergoing three freeze–thaw cycles. The supernatants were then collected for RNA extraction, filtered using a 0.45 µm filter (Biosharp, Hefei, Anhui, China) and stored −80 °C for virus isolation. Viral RNA was extracted using a Viral RNA Kit (Omega Bio-Tek, Inc., Norcross, GA, USA). Reverse transcription was carried out using the GoScript™ Reverse Transcription Mix, Oligo (dT) (Promega Biotech Co., Ltd., Madison, WI, USA) following the manufacturer’s instructions.

The collected samples were detected using reverse transcription polymerase chain reaction (RT-PCR) to amplify the PEDV N gene using a specific pair of primers (Table 3). The RT-PCR reaction was set up in a total volume of 25 µL, containing 12.5 µL of 2 × Green Taq Mix (Vazyme Biotech Co., Ltd., Nanjing, China), 1 µL of each primer (10 µM), 2 µL of cDNA, and 8.5 µL of ddH2O. The thermal cycling program began with a pre-denaturation of 95 °C for 5 min, followed by 40 cycles of 95 °C for 15 s, 48 °C for 30 s, and 72 °C for 30 s, as well as a 5 min extension at 72 °C. The N gene was amplified utilizing a T1000 Thermal Cycler (Bio-Rad Laboratories, Hercules, CA, USA).

### 4.3. Amplification and Sequencing of PEDV S Gene

The S gene was fragmented into three segments (S-1, S-2, and S-3) with partial overlap between each segment. PEDV-positive samples were selected for the amplification of the S gene using the specific segmented primers in Table 3. The RT-PCR system and procedure were the same as described above. Then, the PCR products were analyzed using Power Pac Universal (Bio-Rad Laboratories, Hercules, CA, USA) using 1% agarose gel (Yeasen Biotechnology (Shanghai) Co., Ltd., Shanghai, China) and visualized using the GEL DOC™ XR+ gel documentation system (Bio-Rad Laboratories, Hercules, CA, USA). A gel extraction kit (Omega Bio-Tek, Inc., Norcross, GA, USA) was used for gel extraction, and the PCR products were cloned into the pMD-19T vector (Vazyme Biotech Co., Ltd., Nanjing, China). The cloned fragments were sequenced by Sangon Biotech (Shanghai) Co., Ltd. (Shanghai, China).

### 4.4. Virus Isolation and RT-PCR Identification

Vero cells were seeded in a 12-well plate for virus isolation. The Vero cells were washed twice with PBS, inoculated with 0.5 mL of homogenate supernatant and supplemented with 0.5 mL of DMEM containing 10 µg/mL trypsin (Sigma-Aldrich Trading Co., Ltd., St Louis, MO, USA). The cells were incubated at 37 °C in 5% CO_2_ for 1 h. The inoculum was removed and replaced with DMEM containing 5 µg/mL trypsin. The cells were incubated at 37 °C in 5% CO_2_ for 48–72 h and blindly passaged for seven generations until the cytopathic effect (CPE) was observed. Viral nucleic acids were detected by RT-PCR assays using the cDNA as templates and the primers specific for the PEDV N gene as described above. The PEDV genome was amplified as previously described [46].

### 4.5. TCID_50_ Assay

Vero cells were seeded in 96-well plates and incubated at 37 °C in 5% CO_2_. After 24 h, the cells were visualized under a light microscope to confirm their uniform distribution and confluency of over 80%. A series of ten-fold dilutions were prepared. Then, ten-fold serial virus dilutions were added to each well. Virus titers were calculated via the Reed–Muench method.

### 4.6. IFA

This assay was performed as described previously [47]. Vero cells were seeded onto 14 mm glass cover slips in 24-well plates (NEST, Wuxi, Jiangsu, China). The cells were infected with PEDV. At 24 h post-infection, the cells were washed with PBST three times and treated with 4% paraformaldehyde (PFA) for 30 min at room temperature. The cells were then blocked with blocking buffer (2% bovine serum albumin (BSA) in 0.5% PBST (0.5% Tween-20 in PBS)) at room temperature for 30 min. The, the primary antibody, PEDV-positive serum, pig anti-PEDV (1:100), and mouse anti-double stranded RNA (1:1000) were added and incubated for 45 min. After 1 h, the cells were washed three times with 0.5% PBST, followed by FITC-conjugated goat anti-pig IgG (1:200), Cy3-conjugated goat anti-mouse IgG (1:200), and 4,6-diamidino-2-phenylindole (DAPI, Solarbio Science & Technology Co., Ltd., Beijing, China) at room temperature for 30 min. The cells were then washed five times with PBST. The cells were examined using a confocal microscope (OXFORD instruments, Abingdon, Oxfordshire UK) under a 40× oil objective.

### 4.7. Electron Microscopy Analysis

The virus-containing-supernatants were initially centrifuged at 11,000× *g* for 30 min at 4 °C to remove the cell debris, and then ultracentrifuged at 210,000× *g* for 3 h at 4 °C. The viral particles were resuspended in 500 µL PBS, and then negatively stained with 2% phosphotungstic acid, and examined using transmission electron microscopy (Hitachi, Tokyo, Japan).

### 4.8. Phylogenetic and Recombination Analysis

The best-fit models for the complete genome and spike sequences were selected using Model Finder [48] in PhyloSuite (Version 1.2.2) [49]. Phylogenetic trees were constructed utilizing the maximum likelihood (ML) method in MEGA X (Version 10.1.5) [50]. Bootstrap values were estimated for 1000 replicates. The information about the reference strains of PEDV is displayed in Table 4. Genome recombination analysis was conducted using the Recombination Detection Program v4 (RDP4) [51], which employed seven methods, including RDP (Version 4.1) [52], 3seq (Version 4.1) [53], GENECONV, Chimera (Version 4.1) [54], SiScan (Version 4.1) [55], MaxChi (Version 4.1) [56], and BootScan (Version 4.1) [57]. Simplot (Version 4.1) [58] was applied to display the breakpoint position of the recombination event.

The nucleotide sequence/amino acid homology of the complete genome and spike sequences were initially analyzed by BioAider (Version 1.334) [59] and further analyzed using TBtools (version 2.0) [60]. The amino acid sequences of the spike were aligned with ESPript 3.0 (ESPript 3.x/ENDscript 2.x (ibcp.fr)).

### 4.9. Growth Kinetics

Vero cells were inoculated with PEDV (100 µL of 100 TCID_50_/mL). The cells were incubated at 37 °C in 5% CO_2_. The supernatants were collected at different time points (6, 12, 24, 36, 48, and 72 hpi), and the virus titers were determined via TCID_50_ assay [61]. In brief, the Vero cells were seeded in 96-well plates at a confluence of 90%. Virus supernatants were serially diluted by10-fold, and 100 µL of each dilution was added to individual wells with eight replicates. The wells showing a visible cytopathic effect (CPE) were tallied, and virus titers were determined via the Reed–Muench method.

## 5. Conclusions

In conclusion, we characterized 55 PEDV S genes in seven regions of China during 2020–2022. These sequences all belonged to the GII genotypes, with sporadic prevalence of the GIIa and GIIb strains and wide spread of the GIIc strains. The amino acid alignments showed that unique deletion often occurred within the S1 subunit. We also successfully obtained the PEDV/SC/2022 strain through virus isolation and identification. Through a phylogenetic and recombinant analysis with other representative PEDV strains, a comprehensive understanding of the mutation and recombination of the PEDV strain was achieved.

## Figures and Tables

**Table 1:** The prevalence of PEDV of different sample types during 2020–2022.

Total	Sample Type		Total	PEDV Positive Rate Positive/Sample (%)
	Small Intestine Tissue	Feces		
Sichuan	198	95	293	151/293 (51.54%)
Guizhou	1	4	5	5/5 (100%)
Chongqing	1	0	1	1/1 (100%)
Inner Mongolia	0	16	16	10/16 (62.5%)
Henan	0	14	14	14/14 (100%)
Jiangsu	0	5	5	5/5 (100%)
Guangxi	0	13	13	13/13 (100%)
Total	200	147	347	199/347 (57.35%)

**Table 2:** The information about the PEDV reference sequences used for the alignment of PEDV/SC/2022.

Strain	GenBank No.	Location	Year
CV777	AF353511	Belgium	1977
LZC	EF185992	China	2007
JS2008	KC109141	China	2013
SD-M	JX560761	China	2012
SC1402	KP162057	China	2014
CHHNAY2015	KR809885	China	2015
SNJ_P	MK702008	China	2018
OH851	KJ399978	USA	2014
GER/L00719/2014	LM645058	Germany	2014
OH1414	KJ408801	USA	2014
IA1	KF468753	USA	2013
IA2	KF468754	USA	2013
AH2012	KC210145	China	2012
LWL	MK392335	China	2019
FL2013	KP765609	China	2014
FJzz1	MK288006	China	2011
AJ1102	JX188454	China	2011

**Table 3:** Sequences of primers used in this study.

Primer	Sequence	Lenth (bp)
PEDV-N-F	5′-CACAGATAGTGAGAAAGTGCTTCA-3′	1434
PEDV-N-R	5′-CAGTAATAACAGTGTAATGGCACT-3′	
PEDV-S-1-F	5′-TTTGTGGTTTTTCTAATCATTTGGTCAACG-3′	1659
PEDV-S-1-R	5′-GAACTAAACCCATTGATAGTAGTGTCA-3′	
PEDV-S-2-F	5′-GTCACAATTAATTTCACTGGTC-3′	1716
PEDV-S-2-R	5′-CTGTAGAACATCCGTCTGTAG-3′	
PEDV-S-3-F	5′-GCAGATATAGTCTGTGCAC-3′	1551
PEDV-S-3-R	5′-AGAAGTAGATAAAAACACTGGTG-3′	

**Table 4:** The information about the PEDV reference sequences.

Strain	GenBank No.	Location	Year
CV777	AF353511	Belgium	1977
DR13	DQ862099	South Korea	1999
LZC	EF185992	China	2006
KNU-0802	GU180143	South Korea	2008
KNU-0902	GU180145	South Korea	2009
SM98	GU937797	South Korea	2011
CNU-091222-01	JN184634	South Korea	2009
CH/S	JN547228	China	1986
BJ-2011-1	JN825712	China	2011
CHGD-01	JN980698	China	2011
virulent_DR13	JQ023161	South Korea	2009
attenuated_DR13	JQ023162	South Korea	2002
GD_B	JX088695	China	2012
AJ1102	JX188454	China	2011
LC	JX489155	China	2011
ZJCZ4	JX524137	China	2011
SD-M	JX560761	China	2012
GD-1	JX647847	China	2011
JS2008	KC109141	China	2013
CH/ZMDZY/11	KC196276	China	2011
AH2012	KC210145	China	2012
JS-HZ2012	KC210147	China	2012
USA/Colorado/2013	KF272920	USA	2013
NCH/GDGZ/2012	KF384500	China	2012
USA/Indiana/17846/2013	KF452323	USA	2013
MN	KF468752	USA	2013
IA1	KF468753	USA	2013
IA2	KF468754	USA	2013
CH/JX-1/2013	KF760557	China	2013
CH/YNKM-8/2013	KF761675	China	2013
KPEDV-9	KF898124	South Korea	1997
SHQP/YM/2013	KJ196348	China	2013
OH851	KJ399978	USA	2014
OH1414	KJ408801	USA	2014
KNU-1303	KJ451038	South Korea	2013
KNU-1401	KJ451047	South Korea	2014
KNU-1402	KJ451048	South Korea	2014
CH/JX-2/2013	KJ526096	China	2013
K13JA12-1	KJ539151	South Korea	2014
K14JB01	KJ539154	South Korea	2014
USA/Kansas29/2013	KJ645637	USA: Kansas	2013
USA/Iowa107/2013	KJ645696	USA	2013
MEX/124/2014	KJ645700	Mexico	2014
USA/Minnesota52/2013	KJ645704	USA	2013
VN/VAP1113_1	KJ960179	Viet Nam	2013
GDS01	KM089829	China	2014
KNU-1406-1	KM403155	South Korea	2014
CHM2013	KM887144	China	2013
USA/IA/2013/19321	KM975738	USA	2013
SC1402	KP162057	China	2014
Hawaii/39249/2014	KP688354	USA	2013
SQ2014	KP728470	China	2013
FL2013	KP765609	China	2014
CH/HNYF/14	KP890336	China	2015
15V010/BEL/2015	KR003452	Belgium	2015
FR/001/2014	KR011756	France	2014
PC21A	KR078299	USA	2013
CH/HNQX-3/14	KR095279	China	2015
EAS1	KR610991	Thailand	2015
EAS2	KR610992	Thailand	2014
CBR1	KR610993	Thailand	2014
CH/HNAY/2015	KR809885	China	2015
YN1	KT021227	China	2013
YN15	KT021228	China	2013
CH/HNLH/2015	KT199103	China	2015
CV777	KT323979	China	1998
HUA-14PED96	KT941120	Viet Nam	2014
YC2014	KU252649	China	2014
SLO/JH-11/2015	KU297956	Slovenia	2015
ZJU/G1/2013	KU664503	China	2013
CH/SCCD/2014	KU975389	China	2014
JSLS-1/2015	KX534205	China	2015
KB2013-4	KX580953	China	2013
85-7	KX839246	China	2013
CH/GX/2015/750A	KY793536	China	2015
AVCT12	LC053455	Thailand	2010
IWT-1/JPN/2014	LC063834	Japan	2014
OKN-1/JPN/2013	LC063836	Japan	2013
MYG-1/JPN/2014	LC063838	Japan	2014
MYZ-1/JPN/2013	LC063846	Japan	2013
L00721/GER/2014	LM645057	Germany	2014
GER/L00719/2014	LM645058	Germany	2014
PEDV/GER/L01020-K01_15-10/2015	LT898413	Germany	2015
PEDV/GER/L01014-K01_15-04/2015	LT898420	Germany	2015
PEDV/GER/L00906-K16_14-01/2014	LT898430	Germany	2014
CH/JXJA/2017	MF375374	China	2017
NW8	MF782687	China	2015
PPC 14	MG781192	South Korea	2014
CH/SCZY44/2017	MH061338	China	2017
CH/SCDY523/2018	MH593144	China	2018
JS-A	MH748550	China	2017
FJzz1	MK288006	China	2011
LW/L	MK392335	China	2010
SNJ-P	MK702008	China	2018
PEDV-1556-Valencia-Requena	MN692763	Spain	2014
PEDV-1611-Murcia-Lorca	MN692768	Spain	2015
PEDV-1613-Murcia-Fuentealamo	MN692769	Spain	2015
CH-HNAY-2016	MN893406	China	2016
CH-HNAY-2017	MN893407	China	2017
CH-HNHB-1-2017	MN893408	China	2017
CH-HNHB-2-2017	MN893409	China	2017
CH-HNJY-2017	MN893410	China	2017
CH-HNLH-2016	MN893411	China	2016
CH-HNLH-2017	MN893412	China	2017
CH-HNNY-2017	MN893413	China	2017
CH-HNPDS-1-2018	MN893414	China	2018
CH-HNPDS-2-2018	MN893415	China	2018
CH-HNPY-2016	MN893416	China	2016
CH-HNSMX-2016	MN893417	China	2016
CH-HNXX-2017	MN893418	China	2017
CH-HNXY-1-2017	MN893419	China	2017
CH-HNXY-2-2017	MN893420	China	2017
CH-HNXY-3-2018	MN893421	China	2018
CH-HNZK-1-2017	MN893422	China	2017
CH-HNZK-2-2018	MN893423	China	2018
CH-HNZK-3-2018	MN893424	China	2018
CH-HNZK-4-2017	MN893425	China	2017
CH-HNZMD-2017	MN893426	China	2017
CH-HNZZ-2017	MN893427	China	2017

## Data Availability

All data analyzed during this study are available from the corresponding author on reasonable request.
